# On the Provision of Services With UAVs in Disaster Scenarios: A Two-Stage Stochastic Approach

**DOI:** 10.1007/s43069-022-00127-x

**Published:** 2022-03-02

**Authors:** Gabriella Colajanni, Patrizia Daniele, Daniele Sciacca

**Affiliations:** Department of Mathematics and Computer Science, University of Catania, Catania, Italy

**Keywords:** Unmanned aerial vehicles, Disaster management, Stochastic optimization, Variational inequality

## Abstract

In this paper, we propose a two-stage stochastic optimization model for the provision services in a multi-tiered network, consisting in user or devices on the ground requiring services to controller UAVs in flight. The requested services are executed by a fleet of pre-existing and additional UAVs. The possible occurrence of disaster scenarios and the related uncertainty and severity could cause an unexpected and sudden increase in demand. Hence, the aim of the proposed model is to optimize the management of the pre-existing and additional resources in order to maximize the total profit of service providers and, simultaneously, minimize the expected loss related to a possible unmet demand. A variational approach is proposed, and some numerical examples are performed to validate the effectiveness of our model.

## Introduction

The 5G technology (fifth-generation technology), whose worldwide distribution started in 2019, aims to achieve greater efficiency and versatility in the support of network applications (see [1]). Through the optimization of the use of network resources (see [2]), the definition of virtual subnets, the virtualization of most of the network devices (see [3]), the ability to manage a greater number of devices per unit of surface area, the support of more advanced features in terms of latency to ensure real-time response times, higher data rates and a significant reduction in power consumption, 5G networks are used primarily as general internet service providers, competing with existing ISPs providing networked services, and enable new applications in the Internet of Things, IoT (see [4]), and machine-to-machine areas. In less than a year, 81 operators in 42 countries have launched commercial 5G services and 386 operators in 97 countries have invested in the new networks. The 5G technologies and their features are currently used in all possible fields of application, from retail to education, transportation to entertainment, smart homes to healthcare, agricultural sector to supply chain management (see [5–8]).

Parallel to the advent and growth of 5G networks around the world, COVID-19 pandemic rapidly spread around the world affecting almost all countries and 179 million people, including 3 million deaths (see [9]) and raising enormous health, economic and social challenges (see [10, 11]). The strong containment measures, the nation-wide lockdowns and the social distancing norms, have resulted in increased Internet traffic demands (see [12]) and in the use of digital technologies in the daily lives. Work, education and all human activities have been profoundly changed by the advent of the pandemic and, for instance, educational institutions shift to distance learning to guarantee the education at all levels (see [13]). The need to move all normal activities into a secure and virtual environment is an evidence of the digital acceleration process. In this contest, 5G technologies and their ability to manage a greater number of heterogeneous users or devices and digital traffic can play a vital role to address the wide spectrum of challenges due to COVID-19. (see [14]).

In general, when a disaster occurs, it is plausible to assume that the physical connections that guarantee certain types of services are compromised (for example in the event of an earthquake, landslide, tsunami). In such cases, it is necessary that these types of services are restored quickly or introduced for the first time and the introduction of a 5G network could be the only way to have the provision of services in the event of serious disruptions at ground level, especially if these services are requested by emergency services. The restoration of such services requires that service providers be able to cover the sudden and increasing demand from users or devices on some areas of the ground and this could be challenging and expensive especially if the service providers are totally unprepared for this event. For these reasons, to help the providers to supply the requested services in disasters scenarios, the Unmanned Aerial Vehicles (UAVs) can be very useful (if not even necessary) to monitor hard-to-reach areas and also provide services in rural or energy-deprived areas (see [15] for a survey collecting much earlier work in the intersection of UAVs and communications). The UAVs, such as drones, can be connected to each other and form a real network capable of receiving requests, such as automotive applications, data sharing, wearable health sensors, video calling, smart -traffic control systems, Video Monitoring or video surveillance (see [16] for an extended 5G network slice for video monitoring with a FANET), managing them and executing them through the computer elements they have above (see [17–20] for optimization models for 5G network with UAVs).

Many authors, in their works, are dealt with the use of UAVs in managing a disaster (see [21] for an interesting review of the state-of-the-art optimization approaches in the civil application of drone operations and drone-truck combined operations including construction/infrastructure, agriculture, transportation/logistics, security/disaster management, entertainment/media, etc.). In [22], authors deal with the problem of locating trapped people and routing aid to them after a disaster event. To address the issues associated with the probable failures in the transportation and telecommunications networks, which are often rendered unusable by the disaster at hand, the authors propose two-echelon vehicle routing frameworks for performing these operations using aerial uncrewed autonomous vehicles (UAVs or drones). Specifically, they present two decision frameworks, in which the resulting optimization problem is formulated as a two-echelon vehicle routing problem. The first framework addresses the problem in two stages: providing telecommunications capabilities in the first stage and satisfying the resulting demands in the second. The second framework, on the other hand, addresses the problem as a stochastic emergency aid delivery problem, which uses a two- stage robust optimization model to handle demand uncertainty. Zhao et al. in [23], propose a unified framework of UAV-assisted emergency network in disasters. Particularly, first the trajectory and scheduling of UAV are jointly optimized to provide wireless service to ground devices with surviving BSs and, then, the transceiver design of UAV and establishment of multi-hop ground device-to-device (D2D) communication are studied to extend the wireless coverage of UAV.

Moreover, another aspect of fundamental importance, especially in a disaster situation, is represented by the possibility of adding resources to those already present (see [24]). Such additional resources could be, for instance, new UAVs put into flight and added to the network or they also may consist of increasing the capabilities of pre-existing UAVs. The additional resources make it possible to satisfy a greater quantity of requests (which during disastrous events, as already mentioned above, generally tend to increase or move to zones).

In this paper we propose a two-stage stochastic optimization model, where the first stage is the deterministic stage while the second one depends on the disaster scenario that may occur. Both the preparation stage and the response stage are important in managing a disaster. Particularly, the consideration of uncertainty of possible disaster scenarios and their severity, with the appropriate management of additional resources, could guarantee service providers greater efficiency in their response to a disaster advent.

The paper is organized as follows. In Sect. 2, we present the mathematical network model and derive a two-stage stochastic constrained optimization model whose solutions are obtained through the variational formulation, presented in Sect. 3. Through the Lagrange theory, in Sect. 4, we get an equivalent variational formulation to the one obtained that we will use in the resolution of some numerical examples, contained in Sect. 5, together with a discussion of the results. Finally, Sect. 6 is devoted to the conclusions.

## The Mathematical Model

**Fig. 1:**
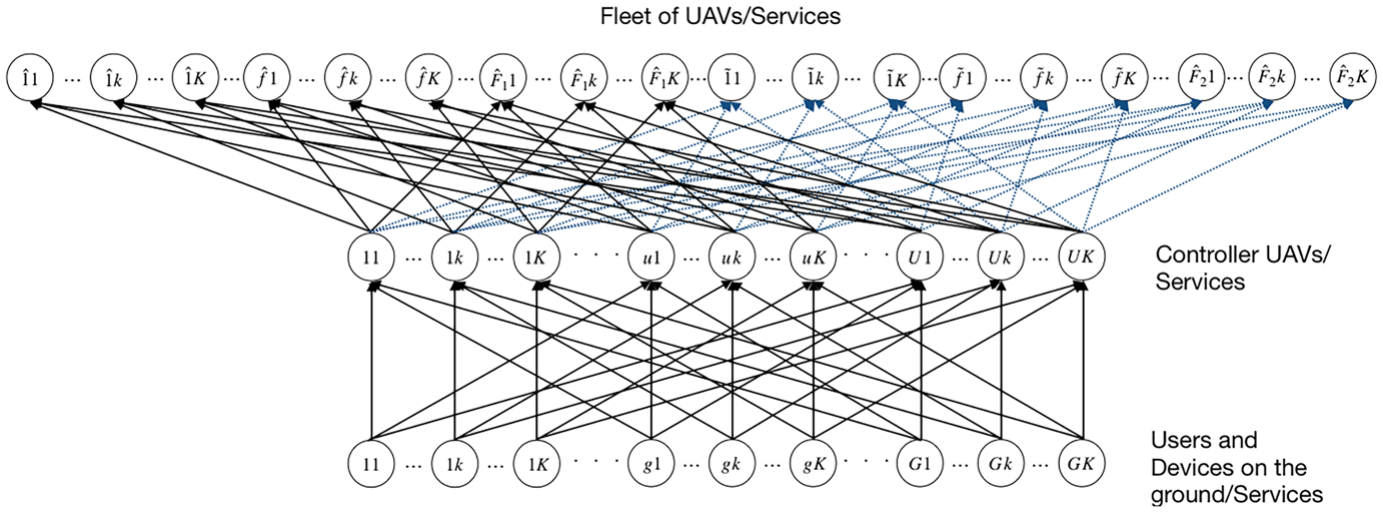
Network Topology

The two-stage stochastic optimization model consists of *G* users or devices on the ground, with a typical one denoted by *g*; *K* types of services, with a generic one denoted by *k*; *U* controller UAVs in flight, with a generic one denoted by *u*; $\hat{F}_1$ pre-existing UAVs, with a generic one denoted by $\hat{f}$ and $\tilde{F}_2$ additional UAVs, with a typical one denoted by $\tilde{f}$. We consider the following sets:

$$\hat{\mathcal {F}}_1=\{\hat{1},\ldots ,\hat{f},\ldots ,\hat{F}_1\},$$

$$\tilde{\mathcal {F}}_2=\{\tilde{1},\ldots ,\tilde{f},\ldots ,\tilde{F}_2\},$$

$$\mathcal {F}_3=\hat{\mathcal {F}}_1 \cup \tilde{\mathcal {F}}_2,$$

that are, respectively, the set of pre-existing UAVs, the set of possible additional UAVs and the set of all the UAVs, where $|\mathcal {F}|=F_3=\hat{F}_1+\tilde{F}_2$.

Services requested by a user or device on the ground are received by the controller UAVs, which are spatially distributed in the considered geographical area, and each controller UAV sends the requests for execution of the services to the fleet of UAVs at the higher level which performs the executions. Moreover, we consider the possibility to add additional drones to the fleet of UAVs and additional capacities to controller UAVs.

The supply chain network, consisting of a fleet of UAVs, controller UAVs and users or devices on the ground, is depicted in Fig. 1.

As mentioned in the Introduction, when a disaster scenario occurs, there may be the need to restore or introduce some services to cope with a possible growth in request. The total unpreparedness of the service providers could make this process expensive, difficult and time-consuming. For these reasons, in this paper, we provide a two-stage stochastic optimization model where the first stage represents the pre-crisis phase and the second one represents the critical phase. In the first stage that represents the preparedness phase, a service provider has to solve a deterministic optimization problem. In the second stage, a service provider considers several scenarios with different probabilities to better face a probable subsequent critical phase, trying to maximize its total profit (defined by the difference between the revenue and the sum of costs and penalties). Hence, the actions that a service provider takes in Stage 2, as soon as the disaster has occurred, depend on the possible scenario and the realization of probabilistic parameters.

Let us consider $\Omega$ the set of all possible disastrous scenarios and $\omega \in \Omega$ a typical disaster scenario.

As previously mentioned, in this model we provide a system stochastic optimization perspective, determining a profit maximization problem for service providers.

The variables and parameters for the model both in Stages 1 and 2 are reported in Tables 1 and 2, respectively.

**Table 1:** Variables for the model

**Notation**	**Variables**
$x_{guk}^1$	the quantity of service *k* requested by user or device *g* on the ground to the controller UAV *u* in Stage 1. We group these quantities, for all services, into the vector $X^{1,1}_{gu}$ and in turn, for all *g* and for all *u*, we group the quantities into the vector $X^{1,1}$.
$x_{u\hat{f}k}^1$	the quantity of service *k* requests sent by the controller UAV *u* to the pre-existing UAV $\hat{f}\in \hat{\mathcal {F}}_{1}$ belonging to the upper tier fleet in Stage 1. We group these quantities, for all *k*, into the vector $X^{2,1}_{u\hat{f}}$ and, in turn, we group these quantities, for all *u*, into the vector $X^{2,1}_{\hat{f}}$. Finally, we group the last vectors, for all $\hat{f}$, into the vector $X^{2,1}$.
$x_{u\tilde{f}k}^1$	the quantity of service *k* requests sent by the controller UAV *u* to the additional UAV $\tilde{f}\in \tilde{\mathcal {F}}_{2}$ which the provider can decide to activate in Stage 1. We group these quantities, for all *k*, into the vector $X^{3,1}_{u\tilde{f}}$ and, in turn, we group these quantities, for all *u*, into the vector $X^{3,1}_{\tilde{f}}$. Finally, we group the last vectors, for all $\tilde{f}$, into the vector $X^{3,1}$.
$\gamma _u^1$	the additional capacity which can be added to controller UAV *u* in Stage 1. We group these quantities into the vector $\Gamma ^1$.
$x_{guk}^{2\omega }$	the quantity of service *k* requested by user or device *g* on the ground to the controller UAV *u* in Stage 2 when scenario $\omega \in \Omega$ occurs. We group these quantities, for all services *k*, into the vector $X^{1,2\omega }_{gu}$ and in turn, for all *g* and for all *u*, we group the quantities into the vector $X^{1,2\omega }$. Finally, we group for all scenarios $\omega \in \Omega$ into the vector $X^{1,2}$.
$x_{u\hat{f}k}^{2\omega }$	the quantity of service *k* requests sent by the controller UAV *u* to the pre-existing UAV $\hat{f}\in \hat{\mathcal {F}}_{1}$ belonging to the upper tier fleet in Stage 2 when scenario $\omega \in \Omega$ occurs. We group these quantities, for all *k*, into the vector $X^{2,2\omega }_{u\hat{f}}$ and, in turn, we group these quantities, for all *u*, into the vector $X^{2,2\omega }_{\hat{f}}$. We group the last vectors, for all $\hat{f}$, into the vector $X^{2,2\omega }$. Finally, we group for all scenarios $\omega \in \Omega$ into the vector $X^{2,2}$.
$x_{u\tilde{f}k}^{2\omega }$	the quantity of service *k* requests sent by the controller UAV *u* to the additional UAV $\tilde{f}\in \tilde{\mathcal {F}}_{2}$ which the provider can decide to activate in Stage 2 when scenario $\omega \in \Omega$ occurs. We group these quantities, for all *k* into the vector $X^{3,2\omega }_{u\tilde{f}}$ and, in turn, we group these quantities, for all *u*, into the vector $X^{3,2\omega }_{\tilde{f}}$. We group the last vectors, for all $\tilde{f}$, into the vector $X^{3,2\omega }$ and, finally, we group for all scenario $\omega \in \Omega$ into the vector $X^{3,2}$.
$\gamma _u^{2\omega }$	the additional capacity which can be added to controller UAV *u* in Stage 2 under scenario $\omega \in \Omega$. We group these quantities, for all *u* into the vector $\Gamma ^{2\omega }$ and for all scenario $\omega \in \Omega$ into the vector $\Gamma ^2$.
$\chi ^1$	the vector $(X^{1,1},X^{2,1},X^{3,1},\Gamma ^{1})$.
$\chi ^2$	the vector $(X^{1,2},X^{2,2},X^{3,2},\Gamma ^{2})$.

**Table 2:** Parameters for the model

**Notation**	**Parameters**
$p_{\omega }$	the probability of disaster scenario $\omega$ in Stage 2, $\omega \in \Omega$.
$R_{gk}^1$	the demand for service (application service or network service) *k* from the user or device *g* on the ground, in Stage 1.
$R_{gk}^{2\omega }$	the demand for service (application service or network service) *k* from the user or device *g* on the ground, in Stage 2 under scenario $\omega$, $\forall \omega \in \Omega$.
$\overline{S}_u$	the maximum capacity related to the controller UAV *u*, that is the maximum number of service requests that the controller UAV *u* is able to manage, $\forall u=1,\ldots ,U$ in both first and second stage.
$s_k$	the execution space requested to perform service *k* in both first and second stage, $\forall k=1, \ldots , K$.
$S_f$	the maximum capacity related to the UAV *f*, $\forall f \in \mathcal {F}_3$, that is the maximum execution space that the (pre-existing or additional) UAV *f* can bear in both first and second stage.
$\overline{\gamma }^1_u$	the maximum additional capacity which can be added to controller UAV *u*, $\forall u=1,\ldots ,U$, in Stage 1.
$\overline{\gamma }^{2\omega }_u$	the maximum additional capacity which can be added to controller UAV *u*, $\forall u=1,\ldots ,U$, in Stage 2 under scenario $\omega$, $\forall \omega \in \Omega$.
$\rho _k^1$	the revenue obtained for a unit of service *k* executed in Stage 1, $\forall k=1,\ldots , K$.
$\rho _k^{2\omega }$	the revenue obtained for a unit of service *k* executed in Stage 2 under scenario $\omega$, $\forall k=1,\ldots , K$, $\forall \omega \in \Omega$;
$\overline{B}^1$	the maximum available budget for additional UAVs at the highest level of the network and additional capacities at the controller UAVs in Stage 1.
$\overline{B}^{2\omega }$	the maximum available budget for additional UAVs at the highest level of the network and additional capacities at the controller UAVs in Stage 2 under scenario $\omega \in \Omega$.
$\beta _k$	the unit penalty encumbered by service providers on the unmet demand of service *k*, $k=1,\dots ,K$.

The cost functions, both in the first and in the second stage, are now described. Let:

- $c_{gu}^1$ be the transmission cost of the service requests from user or device *g* to controller UAV *u* in Stage 1 and let us assume $c_{gu}^1$ is a function of $\displaystyle \sum _{k=1}^K{x_{guk}^1}$:
  $$c_{gu}^1=c_{gu}^1\left( \sum _{k=1}^K{x_{guk}^1}\right) = c_{gu}^1( {X_{gu}^1}), \quad \forall g=1,\ldots ,G,\; \forall u=1,\ldots ,U;$$
- $c_{gu}^{2\omega }$ the transmission cost of the service requests from user or device *g* to controller UAV *u* in Stage 2 under scenario $\omega \in \Omega$ and let us assume $c_{gu}^{2\omega }$ is a function of $\displaystyle \sum _{k=1}^K{x_{guk}^{2\omega }}$:
  $$c_{gu}^{2\omega }=c_{gu}^{2\omega }\left( \sum _{k=1}^K{x_{guk}^{2\omega }}\right) = c_{gu}^{2\omega }( {X_{gu}^{2\omega }}), \quad \forall g=1,\ldots ,G,\; \forall u=1,\ldots ,U,\; \forall \omega \in \Omega ;$$
- $c_{uf}^1$ be the transmission cost of the service requests from controller UAV *u* to any UAV $f \in {\mathcal {F}}_{3}$ in the upper tier fleet (that could be represented by a pre-existing UAV $\hat{f}\in \hat{\mathcal {F}}_{1}$ or an additional UAV $\tilde{f}\in \tilde{\mathcal {F}}_{2}$) in Stage 1 and let us assume $c_{uf}^1$ is a function of $\displaystyle \sum _{k=1}^K{x_{ufk}^1}$:
  $$c_{uf}^1=c_{uf}^1\left( \sum _{k=1}^K{x_{ufk}^1}\right) , \quad \forall u=1,\ldots ,U,\; \forall f \in {\mathcal {F}}_{3},$$
  that is $c_{u\hat{f}}^1=c_{u\hat{f}}^1({X_{u\hat{f}}^{2,1}}), \quad \forall u=1,\ldots ,U,\; \forall \hat{f}\in \hat{{\mathcal {F}}}_{1}$ and $c_{u\tilde{f}}^1=c_{u\tilde{f}}^1({X_{u\tilde{f}}^{3,1}}), \quad \forall u=1,\ldots ,U,\; \forall \tilde{f}\in \tilde{{\mathcal {F}}}_{2}$;
- $c_{uf}^{2\omega }$ be the transmission cost of the service requests from controller UAV *u* to any UAV $f \in {\mathcal {F}}_{3}$ in the upper tier fleet (that could be represented by a pre-existing UAV $\hat{f}\in \hat{\mathcal {F}}_{1}$ or an additional UAV $\tilde{f}\in \tilde{\mathcal {F}}_{2}$) in Stage 2 under scenario $\omega \in \Omega$ and let us assume $c_{uf}^{2\omega }$ is a function of $\displaystyle \sum _{k=1}^K{x_{ufk}^{2\omega }}$:
  $$c_{uf}^{2\omega }=c_{uf}^{2\omega }\left( \sum _{k=1}^K{x_{ufk}^{2\omega }}\right) , \quad \forall u=1,\ldots ,U,\; \forall f \in {\mathcal {F}}_{3}\; \forall \omega \in \Omega ,$$
  that is $c_{u\hat{f}}^{2\omega }=c_{u\hat{f}}^{2\omega }({X_{u\hat{f}}^{2,2\omega }}), \quad \forall u=1,\ldots ,U,\; \forall \hat{f}\in \hat{{\mathcal {F}}}_{1}$ and $c_{u\tilde{f}}^{2\omega }=c_{u\tilde{f}}^{2\omega }({X_{u\tilde{f}}^{3,2\omega }})$, $\forall u=1,\ldots ,U,\; \forall \tilde{f}\in \tilde{{\mathcal {F}}}_{2}$;
- $c_{f}^{(E),1}$ be the execution cost of requested services to the UAV ${f}\in {\mathcal {F}}_{3}$ in Stage 1 and let us assume $c_{f}^{(E),1}$ is a function of the total amount of executed services, $\displaystyle { \sum _{u=1}^{U}\sum _{k=1}^K x_{ufk}^1}$:
  $$\displaystyle c_{f}^{(E),1}=c_{{f}}^{(E),1}\left( { \sum _{u=1}^{U}\sum _{k=1}^K x_{u{f}k}^1} \right) ,$$
  that is $$\displaystyle c_{\hat{f}}^{(E),1}\left( X^{2,1}_{\hat{f}} \right) , c_{\tilde{f}}^{(E),1}\left( X^{3,1}_{\tilde{f}} \right) \quad \forall f \in {\mathcal {F}}_{3}, \;\forall \hat{f} \in \hat{\mathcal {F}}_1,\; \forall \tilde{f}\in \tilde{\mathcal {F}}_2;$$
- $c_{f}^{(E),2\omega }$ be the execution cost of requested services to the UAV ${f}\in {\mathcal {F}}_{3}$ in Stage 2 under scenario $\omega \in \Omega$ and let us assume $c_{f}^{(E),2\omega }$ is a function of the total amount of executed services, $\displaystyle { \sum _{u=1}^{U}\sum _{k=1}^K x_{ufk}^{2\omega }}$:
  $$c_{f}^{(E),2\omega }=c_{{f}}^{(E),2\omega }\left( \sum _{u=1}^{U}\sum _{k=1}^K x_{u{f}k}^{2\omega } \right) ,$$
  that is $$c_{\hat{f}}^{(E),2\omega }\left( X^{2,2\omega }_{\hat{f}}\right) , c_{\tilde{f}}^{(E),2\omega }\left( {X^{3,2\omega }_{\tilde{f}}} \right) ,$$ $$\forall f \in {\mathcal {F}}_{3}, \;\forall \hat{f} \in \hat{\mathcal {F}}_1,\; \forall \tilde{f}\in \tilde{\mathcal {F}}_2,\; \forall \omega \in \Omega ;$$
- $c_{u}^1$ be the cost due to add capacity to controller UAV *u*, $\forall u=1,\ldots ,U$ in Stage 1 and let us assume $c_u^1$ is a function of the additional capacity $\gamma _u^1$, that is:
  $$c_u^1=c_u^1(\gamma ^1_u),\quad \forall u=1,\ldots ,U;$$
- $c_{u}^{2\omega }$ be the cost due to add capacity to controller UAV *u*, $\forall u=1,\ldots ,U$ in Stage 2 when scenario $\omega$ occurs, $\forall \omega \in \Omega$ and let us assume $c_u^{2\omega }$ is a function of the additional capacity in Stage 2 under scenario $\omega$, $\gamma _u^{2\omega }$, that is:
  $$c_u^{2\omega }=c_u^{2\omega }(\gamma ^1_u),\quad \forall u=1,\ldots ,U,\; \forall \omega \in \Omega ;$$
- $c_{\tilde{f}}^1$ be the cost due to add a new UAV $\tilde{f}\in \tilde{\mathcal {F}}_{2}$ at the highest level of the network in Stage 1 and let us assume $c_{\tilde{f}}^1$ is a function of the flow of requests received:
  $$c_{\tilde{f}}^1\left( \sum _{u=1}^U\sum _{k=1}^K x_{u\tilde{f}k}^1\right) =c_{\tilde{f}}^1\left( X_{\tilde{f}}^{3,1}\right) , \quad \forall \tilde{f}\in \tilde{\mathcal {F}}_{2}.$$
  Note that, in this framework, we will suppose that there are not fixed costs to reserving the use of UAVs or they can be considered negligible. Therefore, in the event that no service request is sent to the UAV $\tilde{f}$, we obtain that this function is null ($c_{\tilde{f}}^1(0)=0$).
- $c_{\tilde{f}}^{2\omega }$ be the cost due to add a new UAV $\tilde{f}\in \tilde{\mathcal {F}}_{2}$ at the highest level of the network in Stage 2 under scenario $\omega \in \Omega$ and let us assume $c_{\tilde{f}}^{2\omega }$ is a function of the flow of requests received:
  $$c_{\tilde{f}}^{2\omega }\left( \sum _{u=1}^U\sum _{k=1}^K x_{u\tilde{f}k}^{2\omega }\right) =c_{\tilde{f}}^{2\omega }\left( X_{\tilde{f}}^{3,2\omega }\right) , \quad \forall \tilde{f}\in \tilde{\mathcal {F}}_{2},\; \forall \omega \in \Omega .$$
  As in Stage 1, we will assume that these functions are determined such that they are null in the event that no service request is sent to the UAV $\tilde{f}$ ($c_{\tilde{f}}^{2\omega }(0)=0$).

In this paper the limited flight duration due to the consumption of batteries, that is the limitation that distinguishes UAVs, is also taken into account since we integrated it in the execution cost of requested services and in the maximum capacity of each UAV. Moreover, since we consider the cost of handling requests, at the level of controller UAVs, negligible and we assume to be constant the cost to keep the UAVs *u* in flight, we do not include them in our model, but extension to a more general case is easy. We analyze the system from the point of view of the network and service provider. Therefore, the presented model aims at determining the optimal distributions of services requests flows from users and devices on the ground to the controller UAVs, the optimal distributions of services requests flows from the controller UAVs to the (pre-existing and additional) UAVs belonging to the fleet at the highest level of the network, but also the possible additional capacities to put in each controller UAV.

The objective function to maximize consists of the profit of both the two stages and is given by the total revenue, to which all transmission and execution costs are subtracted, as well as the costs for additional UAVs and to increase the capacities of the controller UAVs are subtracted. Also the expected value of the profit in the second stage is added.

A service provider is faced with the following two-stage stochastic optimization model, in which it seeks to maximize the total expected profit:

1 $$\begin{aligned} {\begin{matrix} &{}\text{ Max }\left\{ \displaystyle \sum _{u=1}^U\sum _{f \in \mathcal {F}_{3}}\sum _{k=1}^K{\rho ^1_{k}x_{ufk}^1}- \sum _{g=1}^G\sum _{u=1}^U c^1_{gu}\left( \sum _{k=1}^K{x^1_{guk}} \right) - \sum _{u=1}^U\sum _{f \in \mathcal {F}_{3}} c^1_{uf}\left( \sum _{k=1}^K{x^1_{ufk}} \right) \right. \\ &{}\left. \displaystyle - \sum _{{f} \in {\mathcal {F}}_3} {c_{{f}}^{(E),1}\left( \sum _{u=1}^U\sum _{k=1}^K{x^1_{u{f}k}} \right) } - \sum _{\tilde{f}\in \tilde{\mathcal {F}}_2}{c^1_{\tilde{f}}\left( \sum _{u=1}^U\sum _{k=1}^K x^1_{u\tilde{f}k}\right) } -\sum _{u=1}^U c_u^1(\gamma _u^1)+\mathbb {E}_{\Omega }[P^2(\chi ^2,\omega )]\right\} \end{matrix}} \end{aligned}$$

subject to:

2 $$\begin{aligned}&\displaystyle \sum _{u=1}^U x^1_{guk}{\ge } R^1_{gk} \quad \forall g=1,\ldots ,G,\; \forall k=1,\ldots ,K, \end{aligned}$$

3 $$\begin{aligned}&\displaystyle \sum _{k=1}^K\sum _{g=1}^G x^1_{guk} \le \overline{S}_{u}{+ \gamma _u^1} \quad \forall u=1,\ldots ,U, \end{aligned}$$

4 $$\begin{aligned}&\displaystyle \sum _{\hat{f}\in \hat{\mathcal {F}}_1}x^1_{u\hat{f}k}+\sum _{\tilde{f}\in \tilde{\mathcal {F}}_2}x^1_{u\tilde{f}k} \le \sum _{g=1}^G x^1_{guk} \quad \forall u=1,\ldots ,U,\; \forall k=1,\ldots ,K, \end{aligned}$$

5 $$\begin{aligned}&\displaystyle \sum _{u=1}^U\sum _{k=1}^K s_k x^1_{ufk} \le S_{f} \quad \forall f\in \mathcal {F}_{3}, \end{aligned}$$

6 $$\begin{aligned}&\displaystyle \sum _{\tilde{f}\in \tilde{\mathcal {F}}_{2}} {c^1_{\tilde{f}}\left( \sum _{u=1}^U\sum _{k=1}^K x^1_{u\tilde{f}k}\right) } +\sum _{u=1}^Uc_u^1(\gamma _{u}^1)\le \overline{B}^1, \end{aligned}$$

7 $$\begin{aligned}&\gamma _u^1\le \overline{\gamma }_u^1, \quad \forall u=1,\ldots ,U,\end{aligned}$$

8 $$\begin{aligned}&\displaystyle x^1_{guk},x^1_{u\hat{f}k}, x^1_{u\tilde{f}k}, {\gamma _u^1} \in \mathbb {R}_+, \forall g,\;\forall u,\;\forall \hat{f},\; \forall \tilde{f}\in \tilde{\mathcal {F}}_2,\; \forall f,\; \forall k. \end{aligned}$$

Constraint (2) represents a conservation law in Stage 1 and establishes that the quantity of service *k* requested by user or device *g* on the ground to all controller UAVs cannot be less than the demand $R_{gk}^1$, in Stage 1 (this allows us not only to meet the demand for Stage 1, but also to prepare for Stage 2).

Constraint (3) establishes that the quantity of service requests that the controller UAV *u* can receive in Stage 1 does not exceed its maximum capacity. Observe that constraint (2) must be verified, therefore, the total capacity of controller UAVs are such as to satisfy the demand of Stage 1 for services from all users and devices.

Constraint (4) states that, in Stage 1, the quantity of service *k* requests sent by the controller UAV *u* to all the pre-existing and additional UAVs is less than or equal to the quantity of service *k* requested by all users or devices on the ground to the controller UAV *u*.

Constraint (5) is a capacity constraint and it establishes that the requests of services that each pre-existing and additional UAV *f*, $\forall f \in \mathcal {F}_3$, can receive in Stage 1 must not exceed the maximum allowed.

Constraint (6) is a budget constraint ([18, 25]) and it states that, in Stage 1, there is a budget limit, $\overline{B}^1$, which represents the maximum available budget for adding new UAVs at the highest level of the network and additional capacities to the controller UAVs.

Constraint (7) states that, in Stage 1, it is possible to add a limited additional capacity to each controller UAV.

Finally, constraints (8) are non-negative constraints in Stage 1.

The last term of the objective function (1) represents the expected value of the profit of service provider in the second stage. Assuming a discrete probability distribution, the expected profit of service provider in the second stage can be written as follows:

9 $$\begin{aligned} \mathbb {E}_{\Omega }[P^2(X^{1,2},X^{2,2},X^{3,2},{\Gamma ^2,}\omega )]=\sum _{\omega \in \Omega }p_{\omega }[P^2(X^{1,2},X^{2,2},X^{3,2},{\Gamma ^2,}\omega )], \end{aligned}$$

namely the weighted sum of the profits in each disaster scenario in Stage 2, $P^2(X^{1,2},X^{2,2},X^{3,2}, {\Gamma ^2,}\omega )$, where the weights are the probabilities $p_{\omega }$ that the scenario $\omega$ occurs, for each $\omega \in \Omega$. This profit depends, in addition to the stochastic parameters and variables previously introduced, also on the unmet demand of services requested on the ground, for which the service provider is forced to pay a penalty cost, i.e.,

10 $$\begin{aligned} \sum _{g=1}^G R_{gk}^{2\omega }-\left( \sum _{u=1}^U\sum _{f\in \mathcal {F}_3}x_{ufk}^{2\omega }+\sum _{u=1}^{U}\sum _{f\in \mathcal {F}_3}x^1_{ufk}-{\sum _{g=1}^G}R^{1}_{gk}\right) . \end{aligned}$$

The profit in Stage 2, in turn, is determined as the solution to the following second stage stochastic maximization problem:

11 $$\begin{aligned} \text{ Max }&\left\{ \displaystyle \sum _{u=1}^U\sum _{f \in \mathcal {F}_{3}}\sum _{k=1}^K\rho ^{2\omega }_{k}x^{2\omega }_{ufk}- \sum _{g=1}^G\sum _{u=1}^U c^{2\omega }_{gu}\left( \sum _{k=1}^K{x^{2\omega }_{guk}} \right) - \sum _{u=1}^U\sum _{f \in \mathcal {F}_{3}} c^{2\omega }_{uf}\left( \sum _{k=1}^K{x^{2\omega }_{ufk}} \right) \right. \nonumber \\&\left. \displaystyle - \sum _{{f} \in {\mathcal {F}}_3} {c_{{f}}^{(E),2\omega }\left( \sum _{u=1}^U\sum _{k=1}^K{x^{2\omega }_{u{f}k}} \right) } - \sum _{\tilde{f}\in \tilde{\mathcal {F}}_2}{c^{2\omega }_{\tilde{f}}\left( \sum _{u=1}^U\sum _{k=1}^K x^{2\omega }_{u\tilde{f}k}\right) }-\sum _{u=1}^U c_u^{2\omega }(\gamma _u^{2\omega })\right. \nonumber \\&\left. -\sum _{k=1}^K\beta _{k}\left[ \sum _{g=1}^G R_{gk}^{2\omega }-\left( \sum _{u=1}^U\sum _{f\in \mathcal {F}_3}x_{ufk}^{2\omega }+\sum _{u=1}^{U}\sum _{f\in \mathcal {F}_3}x^1_{ufk}-{\sum _{g=1}^G}R^{1}_{gk}\right) \right] \right\} \end{aligned}$$

subject to constraints:

12 $$\begin{aligned}&\displaystyle \sum _{u=1}^U x^{2\omega }_{guk}{\le } R^{2\omega }_{gk}{-\left[ \sum _{u=1}^U x^{1}_{guk}-R_{gk}^1\right] }, \quad \forall g,\; \forall k,\;\forall \omega \in \Omega , \end{aligned}$$

13 $$\begin{aligned}&\displaystyle \sum _{k=1}^K\sum _{g=1}^G x^{2\omega }_{guk} \le \overline{S}_{u}{+\gamma _u^{2\omega }}, \quad \forall u\; \forall \omega \in \Omega , \end{aligned}$$

14 $$\begin{aligned}&\displaystyle \sum _{\hat{f}\in \hat{\mathcal {F}}_1}x^{2\omega }_{u\hat{f}k}+\sum _{\tilde{f}\in \tilde{\mathcal {F}}_2}x^{2\omega }_{u\tilde{f}k} \le \sum _{g=1}^G x^{2\omega }_{guk}, \quad \forall u\; \forall k\; \forall \omega \in \Omega , \end{aligned}$$

15 $$\begin{aligned}&\displaystyle \sum _{u=1}^U\sum _{k=1}^K s_k x^{2\omega }_{ufk} \le S_{f}, \quad \forall f\in \mathcal {F}_{3},\; \forall \omega \in \Omega , \end{aligned}$$

16 $$\begin{aligned}&\displaystyle \sum _{\tilde{f}\in \tilde{\mathcal {F}}_{2}} {c^{1}_{\tilde{f}}\left( \sum _{u=1}^U\sum _{k=1}^K x^{1}_{u\tilde{f}k}\right) }+\sum _{u=1}^U c_u^1(\gamma _u^1)\nonumber \\&\qquad \qquad +\sum _{\tilde{f}\in \tilde{\mathcal {F}}_{2}} {c^{2\omega }_{\tilde{f}}\left( \sum _{u=1}^U\sum _{k=1}^K x^{2\omega }_{u\tilde{f}k}\right) }+\sum _{u=1}^U c_u^{2\omega }(\gamma _u^{2\omega }) \le {\overline{B}^{1}+} \overline{B}^{2\omega },\quad \forall \omega , \end{aligned}$$

17 $$\begin{aligned}&\gamma _u^{2\omega }\le \overline{\gamma }_u^{2\omega }, \quad \forall u=1,\ldots ,U,\; \forall \omega \in \Omega , \end{aligned}$$

18 $$\begin{aligned}&\displaystyle x^{2\omega }_{guk},x^{2\omega }_{u\hat{f}k}, x^{2\omega }_{u\tilde{f}k}, \gamma _u^{2\omega } \in \mathbb {R}_+, \forall g,\;\forall u,\;\forall \hat{f},\; \forall \tilde{f}\in \tilde{\mathcal {F}}_2,\; \forall f,\; \forall k,\; \forall \omega . \end{aligned}$$

Constraint (12) ensures that the quantity of service *k* requested by user or device *g* on the ground to all controller UAVs in Stage 2 under scenario $\omega$ does not exceed the demand $R_{gk}^{2\omega }$ minus the amount of services executed and not provided at Stage 1, that is the quantity of services prepared at Stage 1 to supply at Stage 2. Constraints (13)-(18) have the same meaning as the constraints (3)-(8), defined for the first stage, except for the budget constraint (16) which is more complete than the equivalent constraint (6) because it includes the costs for additional UAVs and additional capacities of both Stages 1 and 2 and, hence, the sum of budgets ($\overline{B}^1$ and $\overline{B}^{2\omega }$). Therefore, it is also possible to use part of the unspent budget in Stage 1, during the disastrous event in Stage 2. However, the parameters introduced in Stage 2 differ from the equivalent ones defined in Stage 1, because they are affected by the information about the severity of the disaster event.

Following [26, 27] and the standard stochastic programming theory (see [28, 29]), the first- and second-stage problems can be solved together through a unique maximization problem, that is:

19 $$\begin{aligned} \text{ Max }&\left\{ \displaystyle \sum _{u=1}^U\sum _{f \in \mathcal {F}_{3}}\sum _{k=1}^K{\rho ^1_{k}x_{ufk}^1}- \sum _{g=1}^G\sum _{u=1}^U c^1_{gu}\left( \sum _{k=1}^K{x^1_{guk}} \right) - \sum _{u=1}^U\sum _{f \in \mathcal {F}_{3}} c^1_{uf}\left( \sum _{k=1}^K{x^1_{ufk}} \right) \right. \nonumber \\&\left. \displaystyle - \sum _{{f} \in {\mathcal {F}}_3} {c_{{f}}^{(E),1}\left( \sum _{u=1}^U\sum _{k=1}^K{x^1_{u{f}k}} \right) } - \sum _{\tilde{f}\in \tilde{\mathcal {F}}_2}{c^1_{\tilde{f}}\left( \sum _{u=1}^U\sum _{k=1}^K x^1_{u\tilde{f}k}\right) }-\sum _{u=1}^U c_u^{1}(\gamma _u^1)\right. \nonumber \\&\left. + \sum _{\omega \in \Omega } {p_{\omega }} \left[ \displaystyle \sum _{u=1}^U\sum _{f \in \mathcal {F}_{3}}\sum _{k=1}^K\rho ^{2\omega }_{k}x^{2\omega }_{ufk}- \sum _{g=1}^G\sum _{u=1}^U c^{2\omega }_{gu}\left( \sum _{k=1}^K{x^{2\omega }_{guk}} \right) - \sum _{u=1}^U\sum _{f \in \mathcal {F}_{3}} c^{2\omega }_{uf}\left( \sum _{k=1}^K{x^{2\omega }_{ufk}} \right) \right. \right. \nonumber \\&\left. \left. \displaystyle - \sum _{{f} \in {\mathcal {F}}_3} {c_{{f}}^{(E),2\omega }\left( \sum _{u=1}^U\sum _{k=1}^K{x^{2\omega }_{u{f}k}} \right) } - \sum _{\tilde{f}\in \tilde{\mathcal {F}}_2}{c^{2\omega }_{\tilde{f}}\left( \sum _{u=1}^U\sum _{k=1}^K x^{2\omega }_{u\tilde{f}k}\right) }-\sum _{u=}^U c_u^{2\omega }(\gamma _u^{2\omega })\right. \right. \nonumber \\&\left. \left. -\sum _{k=1}^K\beta _{k}\left( \sum _{g=1}^G R_{gk}^{2\omega }-\left( \sum _{u=1}^U\sum _{f\in \mathcal {F}_3}x_{ufk}^{2\omega }+\sum _{u=1}^{U}\sum _{f\in \mathcal {F}_3}x^1_{ufk}-{\sum _{g=1}^G}R^{1}_{gk}\right) \right) \right] \right\} \end{aligned}$$

subject to constraints (2)-(8) and (12)-(18).

## Variational Formulation

In this section, we derive a variational inequality formulation ([30]) of the stochastic maximization problem described in the previous section. We assume that all the involved cost functions are continuously differentiable and convex. We have the following result.

### Theorem 1

A vector $\chi ^*\in \mathbb {K}$ is a solution to the stochastic maximization problem (19);(2)-(8);(12)-(18) if and only if it satisfies the variational inequality: Find $\chi ^*=(\chi ^{1*},\chi ^{2*})\in \mathbb {K}$ such that:

$$\begin{aligned}&\sum _{g=1}^G\sum _{u=1}^U\sum _{k=1}^K\left[ \frac{\partial c_{gu}^1(X^{1,1*}_{gu})}{\partial x_{guk}^1}\right] \times (x_{guk}^1-x^{1*}_{guk})\\&+\sum _{u=1}^U\sum _{\hat{f}\in \hat{\mathcal {F}}_1}\sum _{k=1}^K\left[ \frac{\partial c_{u\hat{f}}^1(X^{2,1*}_{u\hat{f}})}{\partial x_{u\hat{f}k}^1} {+\frac{\partial c_{\hat{f}}^{(E),1}\left( {X_{\hat{f}}^{2,1*}} \right) }{\partial x_{u\hat{f}k}^1}} -\rho ^1_{k}-\beta _k\right] \times (x_{u\hat{f}k}^1-x^{1*}_{u\hat{f}k})\\&+\sum _{u=1}^U\sum _{\tilde{f}\in \tilde{\mathcal {F}}_2}\sum _{k=1}^K\left[ \frac{\partial c^1_{u\tilde{f}}(X^{3,1*}_{u\tilde{f}})}{\partial x_{u\tilde{f}k}^1} {+\frac{\partial c_{\tilde{f}}^{(E),1}\left( {X_{\tilde{f}}^{3,1*}} \right) }{\partial x^1_{u\tilde{f}k}}} +\frac{\partial c^1_{\tilde{f}}(X_{\tilde{f}}^{3,1*})}{\partial x_{u\tilde{f}k}^1}-\rho _k^1-\beta _k\right] \times (x^1_{u\tilde{f}k}-x^{1*}_{u\tilde{f}k})\\&+\sum _{u=1}^U\left[ \frac{\partial c_u^1(\gamma _u^{1*})}{\partial \gamma _u^1}\right] \times (\gamma _u^1-\gamma _u^{1*})\\&+\sum _{\omega \in \Omega }p_{\omega }\sum _{g=1}^G\sum _{u=1}^U\sum _{k=1}^K\left[ \frac{\partial c_{gu}^{2\omega }(X^{1,{2\omega }*}_{gu})}{\partial x_{guk}^{2\omega }}\right] \times (x_{guk}^{2\omega }-x^{2\omega *}_{guk})\\&+\sum _{\omega \in \Omega }p_{\omega }\sum _{u=1}^U\sum _{\hat{f}\in \hat{\mathcal {F}}_1}\sum _{k=1}^K\left[ \frac{\partial c_{u\hat{f}}^{2\omega }(X^{2,2\omega *}_{u\hat{f}})}{\partial x_{u\hat{f}k}^{2\omega }} {+\frac{\partial c_{\hat{f}}^{(E),2\omega }\left( {X_{\hat{f}}^{2,2\omega *}} \right) }{\partial x_{u\hat{f}k}^{2\omega }}} -\rho ^{2\omega }_{k}-\beta _k\right] \times (x_{u\hat{f}k}^{2\omega }-x^{2\omega *}_{u\hat{f}k})\\&+\sum _{\omega \in \Omega }p_{\omega }\sum _{u=1}^U\sum _{\tilde{f}\in \tilde{\mathcal {F}}_2}\sum _{k=1}^K\left[ \frac{\partial c^{2\omega }_{u\tilde{f}}(X^{3,2\omega *}_{u\tilde{f}})}{\partial x_{u\tilde{f}k}^{2\omega }}+\frac{\partial c_{\tilde{f}}^{(E),2\omega }\left( X_{\tilde{f}}^{3,2\omega *} \right) }{\partial x^{2\omega }_{u\tilde{f}k}}\right. \\&\left. \qquad \qquad \quad \qquad \qquad \qquad \qquad +\frac{\partial c^{2\omega }_{\tilde{f}}(X_{\tilde{f}}^{3,2\omega *})}{\partial x_{u\tilde{f}k}^{2\omega }}-\rho _k^{2\omega }-\beta _k\right] \times (x^{2\omega }_{u\tilde{f}k}-x^{2\omega *}_{u\tilde{f}k})\\&+\sum _{\omega \in \Omega }p_{\omega }\sum _{u=1}^U\left[ \frac{\partial c_u^{2\omega }(\gamma _u^{2\omega *})}{\partial \gamma _u^{2\omega }}\right] \times (\gamma _u^{2\omega }-\gamma _u^{2\omega *})\ge 0, \end{aligned}$$

20 $$\begin{aligned} \forall \chi =(\chi ^1,\chi ^2)\in \mathbb {K}, \end{aligned}$$

where

21 $$\begin{aligned} \mathbb {K}:=\Big \{\chi =(\chi ^1,\chi ^2)\in \mathbb {R}_+^{U(1+|\Omega |)[(G+F_3)K+1]} \text{ such } \text{ that: } (2)-(8) \text{ and } (12)-(18) \text{ hold }\Big \} \end{aligned}$$

is the feasible set of the problem.

The previous theorem allows us to utilize the well-known variational inequality theory to obtain the optimal solutions of the problem (see [30]).

We can put variational inequality (20) into standard form, that is: find a vector $X^*\in \mathcal {K}$ such that:

22 $$\begin{aligned} \langle F(X^*),X-X^*\rangle \ge 0,\quad \forall X\in \mathcal {K}, \end{aligned}$$

where $\mathcal {K}$ is a closed, convex set. In this regard, we set $X=\chi$, $F(X)=(F^i(X))_{i=1,\dots ,8}$ and $\mathcal {K}\equiv \mathbb {K}$, where:

$$F^1_{guk}(X)\equiv \left[ \frac{\partial c_{gu}^1(X^{1,1}_{gu})}{\partial x_{guk}^1}\right] ,\quad \forall g,u,k,$$

$$F^2_{u\hat{f}k}(X)\equiv \left[ \frac{\partial c_{u\hat{f}}^1(X^{2,1}_{u\hat{f}})}{\partial x_{u\hat{f}k}^1} {+\frac{\partial c_{\hat{f}}^{1,(E)}\left( {X_{\hat{f}}^{2,1}} \right) }{\partial x_{u\hat{f}k}^1}} -\rho ^1_{k}-\beta _k\right] ,\quad \forall u,\hat{f},k,$$

$$F^3_{u\tilde{f}k}\equiv \left[ \frac{\partial c^1_{u\tilde{f}}(X^{3,1}_{u\tilde{f}})}{\partial x_{u\tilde{f}k}^1} {+\frac{\partial c_{\tilde{f}}^{(E),1}\left( {X_{\tilde{f}}^{3,1}} \right) }{\partial x^1_{u\tilde{f}k}}} +\frac{\partial c^1_{\tilde{f}}(X_{\tilde{f}}^{3,1})}{\partial x_{u\tilde{f}k}^1}-\rho _k^1-\beta _k\right] ,\quad \forall u\tilde{f},k,$$

$$F^4_u\equiv \left[ \frac{\partial c_u^1(\gamma _u^1)}{\partial \gamma _{u}^1}\right] ,\quad \forall u,$$

$$F^{5,\omega }_{guk}\equiv p_{\omega }\left[ \frac{\partial c_{gu}^{2\omega }(X^{1,{2\omega }}_{gu})}{\partial x_{guk}^{2\omega }}\right] ,\quad \forall g,u,k,\omega$$

$$F^{6,\omega }_{u\hat{f}k}\equiv p_{\omega }\left[ \frac{\partial c_{u\hat{f}}^{2\omega }(X^{2,2\omega }_{u\hat{f}})}{\partial x_{u\hat{f}k}^{2\omega }} {+\frac{\partial c_{\hat{f}}^{(E),2\omega }\left( {X_{\hat{f}}^{2,2\omega }} \right) }{\partial x_{u\hat{f}k}^{2\omega }}} -\rho ^{2\omega }_{k}-\beta _k\right] ,\quad \forall u,\hat{f},k,\omega ,$$

$$F^{7,\omega }_{u\tilde{f}k}\equiv p_{\omega }\left[ \frac{\partial c^{2\omega }_{u\tilde{f}}(X^{3,2\omega }_{u\tilde{f}})}{\partial x_{u\tilde{f}k}^{2\omega }} {+\frac{\partial c_{\tilde{f}}^{(E),2\omega }\left( {X_{\tilde{f}}^{3,2\omega }} \right) }{\partial x^{2\omega }_{u\tilde{f}k}}} +\frac{\partial c^{2\omega }_{\tilde{f}}(X_{\tilde{f}}^{3,2\omega })}{\partial x_{u\tilde{f}k}^{2\omega }}-\rho _k^{2\omega }-\beta _k\right] ,\quad \forall u,\tilde{f},k,\omega ,$$

23 $$\begin{aligned} F^{8,\omega }_{u}\equiv p_{\omega }\left[ \frac{\partial c_u^{2\omega }(\gamma _u^{2\omega })}{\partial \gamma _{u}^1}\right] ,\quad \forall u,\omega . \end{aligned}$$

We note that, under the imposed assumptions for cost functions, *F*(*X*) is a continuous function. Moreover, the feasible set, $\mathcal {K}$, is compact. Hence, from the classical theory of variational inequalities (see [31]), a solution to variational inequality (20) or, equivalently to variational inequality (22), is guaranteed to exist. Moreover, a uniqueness result is ensured since the strictly monotonicity of function *F*(*X*) is guaranteed by the following:

### Theorem 2

Let all the involved time and cost functions strictly convex in their variables. Then, the vector function *F*(*X*), defined by (23), is strictly monotone, i.e.,

$$\langle F(X)-F(Y),X-Y\rangle \ge 0,\quad \forall X,Y\in \mathcal {K},\ X\ne Y.$$

### Proof

Let $X_1=(X_1^{1,1},X^{2,1}_1,X^{3,1}_1,\Gamma _1^1,X^{1,2}_1,X^{2,2}_1,X^{3,2}_1,\Gamma _{1}^2)\in \mathcal {K}$ and $X_2=(X_2^{1,1},X^{2,1}_2,$ $X^{3,1}_2,\Gamma _2^1,X^{1,2}_2,X^{2,2}_2,X^{3,2}_2,\Gamma _{2}^2)\in \mathcal {K}$ be two admissible vectors such that $X_1\ne X_2$. We evaluate the quantity:

24 $$\begin{aligned} \langle F(X_1)-F(X_2),X_1-X_2\rangle =\sum _{i=1}^8F^i(X)(X^i_1-X^i_2). \end{aligned}$$

Since all the involved cost functions are strictly convex, each of the eight terms in (24) are strictly greater than 0, if $X_1\ne X_2$. Hence, *F*(*X*) is a strictly monotone function.

## Lagrange Theory and Alternative Variational Inequality Formulation

In this section, we investigate the Lagrange theory associated with variational inequality (20) (see, for instance, [32–36] for an application of the Lagrange theory to various network models). Particularly, due to the non-linearity budget constraints (6) and (16), we relax such constraints into the objective function by associating to them the Lagrange multipliers. We also derive an alternative variational inequality to the one in (20) that we will use in the next section, devoted to numerical examples.

Let $\lambda ^1\in \mathbb {R}_+$ be the Lagrange multiplier associated with budget constraint (6). Likewise, let $\lambda ^{2\omega }\in \mathbb {R}_+$ be the Lagrange multipliers associated with budget constraints (16), for each scenario $\omega \in \Omega$. Let $\lambda ^2=(\lambda ^{2\omega })_{\omega \in \Omega }\in \mathbb {R}_+^{|\Omega |}$.

We set:

25 $$\begin{aligned} a^1(X)=\displaystyle \sum _{\tilde{f}\in \tilde{\mathcal {F}}_{2}} {c^1_{\tilde{f}}\left( \sum _{u=1}^U\sum _{k=1}^K x^1_{u\tilde{f}k}\right) } +\sum _{u=1}^Uc_u^1(\gamma _{u}^1)-\overline{B}^1, \end{aligned}$$

26 $$\begin{aligned} {\begin{matrix} b^{2\omega }(X)=&{}\displaystyle \sum _{\tilde{f}\in \tilde{\mathcal {F}}_{2}} {c^{1}_{\tilde{f}}\left( \sum _{u=1}^U\sum _{k=1}^K x^{1}_{u\tilde{f}k}\right) }+\sum _{u=1}^U c_u^1(\gamma _u^1)+ \sum _{\tilde{f}\in \tilde{\mathcal {F}}_{2}} {c^{2\omega }_{\tilde{f}}\left( \sum _{u=1}^U\sum _{k=1}^K x^{2\omega }_{u\tilde{f}k}\right) }\\ &{}+\sum _{u=1}^U c_u^{2\omega }(\gamma _u^{2\omega })-\overline{B}^{1}- \overline{B}^{2\omega },\quad \forall \omega , \end{matrix}} \end{aligned}$$

and we define:

$${\begin{aligned} &{}V(X)=\sum _{g=1}^G\sum _{u=1}^U\sum _{k=1}^K\left[ \frac{\partial c_{gu}^1(X^{1,1*}_{gu})}{\partial x_{guk}^1}\right] \times (x_{guk}^1-x^{1*}_{guk})\\ &{}+\sum _{u=1}^U\sum _{\hat{f}\in \hat{\mathcal {F}}_1}\sum _{k=1}^K\left[ \frac{\partial c_{u\hat{f}}^1(X^{2,1*}_{u\hat{f}})}{\partial x_{u\hat{f}k}^1} {+\frac{\partial c_{\hat{f}}^{(E),1}\left( {X_{\hat{f}}^{2,1*}} \right) }{\partial x_{u\hat{f}k}^1}} -\rho ^1_{k}-\beta _k\right] \times (x_{u\hat{f}k}^1-x^{1*}_{u\hat{f}k})\\ &{}+\sum _{u=1}^U\sum _{\tilde{f}\in \tilde{\mathcal {F}}_2}\sum _{k=1}^K\left[ \frac{\partial c^1_{u\tilde{f}}(X^{3,1*}_{u\tilde{f}})}{\partial x_{u\tilde{f}k}^1} {+\frac{\partial c_{\tilde{f}}^{(E),1}\left( {X_{\tilde{f}}^{3,1*}} \right) }{\partial x^1_{u\tilde{f}k}}} +\frac{\partial c^1_{\tilde{f}}(X_{\tilde{f}}^{3,1*})}{\partial x_{u\tilde{f}k}^1}-\rho _k^1-\beta _k\right] \times (x^1_{u\tilde{f}k}-x^{1*}_{u\tilde{f}k})\\ &{}+\sum _{u=1}^U\left[ \frac{\partial c_u^1(\gamma _u^{1*})}{\partial \gamma _u^1}\right] \times (\gamma _u^1-\gamma _u^{1*})\\ &{}+\sum _{\omega \in \Omega }p_{\omega }\sum _{g=1}^G\sum _{u=1}^U\sum _{k=1}^K\left[ \frac{\partial c_{gu}^{2\omega }(X^{1,{2\omega }*}_{gu})}{\partial x_{guk}^{2\omega }}\right] \times (x_{guk}^{2\omega }-x^{2\omega *}_{guk})\\ &{}+\sum _{\omega \in \Omega }p_{\omega }\sum _{u=1}^U\sum _{\hat{f}\in \hat{\mathcal {F}}_1}\sum _{k=1}^K\left[ \frac{\partial c_{u\hat{f}}^{2\omega }(X^{2,2\omega *}_{u\hat{f}})}{\partial x_{u\hat{f}k}^{2\omega }} {+\frac{\partial c_{\hat{f}}^{(E),2\omega }\left( {X_{\hat{f}}^{2,2\omega *}} \right) }{\partial x_{u\hat{f}k}^{2\omega }}} -\rho ^{2\omega }_{k}-\beta _k\right] \times (x_{u\hat{f}k}^{2\omega }-x^{2\omega *}_{u\hat{f}k})\\ &{}+\sum _{\omega \in \Omega }p_{\omega }\sum _{u=1}^U\sum _{\tilde{f}\in \tilde{\mathcal {F}}_2}\sum _{k=1}^K\left[ \frac{\partial c^{2\omega }_{u\tilde{f}}(X^{3,2\omega *}_{u\tilde{f}})}{\partial x_{u\tilde{f}k}^{2\omega }} {+\frac{\partial c_{\tilde{f}}^{(E),2\omega }\left( {X_{\tilde{f}}^{3,2\omega *}} \right) }{\partial x^{2\omega }_{u\tilde{f}k}}} \right. \\ &{}\left. \qquad \qquad \qquad \qquad \qquad +\frac{\partial c^{2\omega }_{\tilde{f}}(X_{\tilde{f}}^{3,2\omega *})}{\partial x_{u\tilde{f}k}^{2\omega }}-\rho _k^{2\omega }-\beta _k\right] \times (x^{2\omega }_{u\tilde{f}k}-x^{2\omega *}_{u\tilde{f}k})\\ &{}+\sum _{\omega \in \Omega }p_{\omega }\sum _{u=1}^U\left[ \frac{\partial c_u^{2\omega }(\gamma _u^{2\omega *})}{\partial \gamma _u^{2\omega }}\right] \times (\gamma _u^{2\omega }-\gamma _u^{2\omega *}). \end{aligned}}$$

Let us consider the Lagrange function:

27 $$\begin{aligned} \displaystyle \mathcal {L}(X,\lambda ^1,\lambda ^{2})=V(X)+a^1(X)\lambda ^1+\sum _{\omega \in \Omega }b^{2\omega }(X)\lambda ^{2\omega },\quad \forall X\in \mathcal {K},\ \forall \lambda ^1\in \mathbb {R}_+,\ \forall \lambda ^{2\omega }\in \mathbb {R}_+. \end{aligned}$$

Variational inequality (20) is equivalent to the following problem

28 $$\begin{aligned} \min _{X\in \mathcal {K}} V(X)=0. \end{aligned}$$

This equivalence is justified because we have

$$V(X)\ge 0 \text{ in } \mathcal {K} \text{ and } \min _{X\in \mathcal {K}} V(X)=V(X^{*})=0.$$

We recall that all the involved functions are convex and continuously differentiable. Moreover, since $\mathcal {K}$ is convex and the Slater condition is satisfied, if $X^{*}$ is a minimal solution to problem (28), there exist $\lambda ^{1*}\in \mathbb {R}_+$ and $\lambda ^{2*}\in \mathbb {R}_+^{|\Omega |}$, such that the vector $(X^{*},\lambda ^{1*},\lambda ^{2*})$ is a saddle point of the Lagrange function (27):

29 $$\begin{aligned} \mathcal {L}(X^*,\lambda ^1,\lambda ^2)\le \mathcal {L}(X^*,\lambda ^{1*},\lambda ^{2*})\le \mathcal {L}(X,\lambda ^{1*},\lambda ^{2*}) \end{aligned}$$

and

30 $$\begin{aligned}&a^{1}(X^*)\lambda ^{1*}=0;\nonumber \\&b^{2\omega }(X^*)\lambda ^{2\omega *}=0,\quad \forall \omega \in \Omega . \end{aligned}$$

The right-hand side of (29) ensures that $X^*$ is a minimal point of the function $\mathcal {L}(X,\lambda ^{1*},\lambda ^{2*})$. Hence, for all $u,\ \tilde{f},\ k$ and for all $\omega \in \Omega$, we have:

31 $$\begin{aligned} \frac{\partial \mathcal {L}(X^*,\lambda ^{1*},\lambda ^{2*})}{\partial x^1_{u\tilde{f}k}}&=\left[ \frac{\partial c^1_{u\tilde{f}}(X^{3,1*}_{u\tilde{f}})}{\partial x_{u\tilde{f}k}^1} {+\frac{\partial c_{\tilde{f}}^{(E),1}\left( {X_{\tilde{f}}^{3,1*}} \right) }{\partial x^1_{u\tilde{f}k}}} +\frac{\partial c^1_{\tilde{f}}(X_{\tilde{f}}^{3,1*})}{\partial x_{u\tilde{f}k}^1}\right. \nonumber \\&\left. \qquad -\rho _k^1-\beta _k{+}(\lambda ^{1*}+\sum _{\omega \in \Omega }\lambda ^{2\omega *})\frac{\partial c_{\tilde{f}}^1(X^{3,1*})}{\partial x_{u\tilde{f}k}^1}\right] =0, \end{aligned}$$

32 $$\begin{aligned} \frac{\partial \mathcal {L}(X^*,\lambda ^{1*},\lambda ^{2*})}{\partial \gamma _u^1}=\left[ \frac{\partial c_{u}^1(\gamma _u^{1*})}{\partial \gamma _u^1}{+}(\lambda ^{1*}+\sum _{\omega \in \Omega }\lambda ^{2\omega *})\frac{\partial c_{u}^1(\gamma _u^{1*})}{\partial \gamma _u^1}\right] =0, \end{aligned}$$

33 $$\begin{aligned} \frac{\partial \mathcal {L}(X^*,\lambda ^{1*},\lambda ^{2*})}{\partial x^{2\omega }_{u\tilde{f}k}}&=\left[ \frac{\partial c^{2\omega }_{u\tilde{f}}(X^{3,2\omega *}_{u\tilde{f}})}{\partial x_{u\tilde{f}k}^{2\omega }}{+\frac{\partial c_{\tilde{f}}^{(E),2\omega }\left( {X_{\tilde{f}}^{3,2\omega *}} \right) }{\partial x^{2\omega }_{u\tilde{f}k}}} +\frac{\partial c^{2\omega }_{\tilde{f}}(X_{\tilde{f}}^{3,2\omega *})}{\partial x_{u\tilde{f}k}^{2\omega }}\right. \nonumber \\&\left. \qquad -\rho _k^{2\omega }-\beta _k{+}\lambda ^{2\omega *}\frac{\partial c^{2\omega }_{\tilde{f}}(X_{\tilde{f}}^{3,2\omega *})}{\partial x_{u\tilde{f}k}^{2\omega }}\right] =0, \end{aligned}$$

34 $$\begin{aligned} \frac{\partial \mathcal {L}(X^*,\lambda ^{1*},\lambda ^{2*})}{\partial \gamma ^{2\omega }_u}=\left[ \frac{\partial c_u^{2\omega }(\gamma _u^{2\omega *})}{\partial \gamma _u^{2\omega }}{+}\lambda ^{2\omega *}\frac{\partial c_u^{2\omega }(\gamma _u^{2\omega *})}{\partial \gamma _u^{2\omega }}\right] , \end{aligned}$$

together with conditions (30).

Equations (31)-(34) and conditions (30) allow us to provide an alternative variational inequality formulation to the one in (20).

### Theorem 3

Variational inequality (20) is equivalent to the following one: Determine $(X^*,\lambda ^{1*},\lambda ^{2*})\in \mathbb {K}$ such that

$${\begin{aligned} &{}\sum _{g=1}^G\sum _{u=1}^U\sum _{k=1}^K\left[ \frac{\partial c_{gu}^1(X^{1,1*}_{gu})}{\partial x_{guk}^1}\right] \times (x_{guk}^1-x^{1*}_{guk})\\ &{}+\sum _{u=1}^U\sum _{\hat{f}\in \hat{\mathcal {F}}_1}\sum _{k=1}^K\left[ \frac{\partial c_{u\hat{f}}^1(X^{2,1*}_{u\hat{f}})}{\partial x_{u\hat{f}k}^1} {+\frac{\partial c_{\hat{f}}^{(E),1}\left( {X_{\hat{f}}^{2,1*}} \right) }{\partial x_{u\hat{f}k}^1}} -\rho ^1_{k}-\beta _k\right] \times (x_{u\hat{f}k}^1-x^{1*}_{u\hat{f}k})\\ &{}+\sum _{u=1}^U\sum _{\tilde{f}\in \tilde{\mathcal {F}}_2}\sum _{k=1}^K\left[ \frac{\partial c^1_{u\tilde{f}}(X^{3,1*}_{u\tilde{f}})}{\partial x_{u\tilde{f}k}^1} {+\frac{\partial c_{\tilde{f}}^{(E),1}\left( {X_{\tilde{f}}^{3,1*}} \right) }{\partial x^1_{u\tilde{f}k}}} +\frac{\partial c^1_{\tilde{f}}(X_{\tilde{f}}^{3,1*})}{\partial x_{u\tilde{f}k}^1}-\rho _k^1-\beta _k+\right. \\ &{}\left. \quad \qquad \qquad \qquad (\lambda ^{1*}+{\sum _{\omega \in \Omega }}\lambda ^{2\omega *})\frac{\partial c_{\tilde{f}}^1(X^{3,1*})}{\partial x_{u\tilde{f}k}^1}\right] \times (x^1_{u\tilde{f}k}-x^{1*}_{u\tilde{f}k})\\ &{}+\sum _{u=1}^U\left[ (1+\lambda ^{1*}+{\sum _{\omega \in \Omega }}\lambda ^{2\omega *})\frac{\partial c_{u}^1(\gamma _u^{1*})}{\partial \gamma _u^1}\right] \times (\gamma _u^1-\gamma _u^{1*}) \\ &{}+\sum _{\omega \in \Omega }p_{\omega }\sum _{g=1}^G\sum _{u=1}^U\sum _{k=1}^K\left[ \frac{\partial c_{gu}^{2\omega }(X^{1,{2\omega }*}_{gu})}{\partial x_{guk}^{2\omega }}\right] \times (x_{guk}^{2\omega }-x^{2\omega *}_{guk})\\ &{}+\sum _{\omega \in \Omega }p_{\omega }\sum _{u=1}^U\sum _{\hat{f}\in \hat{\mathcal {F}}_1}\sum _{k=1}^K\left[ \frac{\partial c_{u\hat{f}}^{2\omega }(X^{2,2\omega *}_{u\hat{f}})}{\partial x_{u\hat{f}k}^{2\omega }} {+\frac{\partial c_{\hat{f}}^{(E),2\omega }\left( {X_{\hat{f}}^{2,2\omega *}} \right) }{\partial x_{u\hat{f}k}^{2\omega }}} -\rho ^{2\omega }_{k}-\beta _k\right] \times (x_{u\hat{f}k}^{2\omega }-x^{2\omega *}_{u\hat{f}k})\\ &{}+\sum _{\omega \in \Omega }p_{\omega }\sum _{u=1}^U\sum _{\tilde{f}\in \tilde{\mathcal {F}}_2}\sum _{k=1}^K\left[ \frac{\partial c^{2\omega }_{u\tilde{f}}(X^{3,2\omega *}_{u\tilde{f}})}{\partial x_{u\tilde{f}k}^{2\omega }} {+\frac{\partial c_{\tilde{f}}^{(E),2\omega }\left( {X_{\tilde{f}}^{3,2\omega *}} \right) }{\partial x^{2\omega }_{u\tilde{f}k}}} +\frac{\partial c^{2\omega }_{\tilde{f}}(X_{\tilde{f}}^{3,2\omega *})}{\partial x_{u\tilde{f}k}^{2\omega }}-\rho _k^{2\omega }-\beta _k\right. \\ &{}\left. \qquad \qquad \qquad \qquad \qquad {+}\lambda ^{2\omega *}\frac{\partial c^{2\omega }_{\tilde{f}}(X_{\tilde{f}}^{3,2\omega *})}{\partial x_{u\tilde{f}k}^{2\omega }}\right] \times (x^{2\omega }_{u\tilde{f}k}-x^{2\omega *}_{u\tilde{f}k})\\ &{}+\sum _{\omega \in \Omega }p_{\omega }\sum _{u=1}^U\left[ \frac{\partial c_u^{2\omega }(\gamma _u^{2\omega *})}{\partial \gamma _u^{2\omega }}{+}\lambda ^{2\omega *}\frac{\partial c_u^{2\omega }(\gamma _u^{2\omega *})}{\partial \gamma _u^{2\omega }}\right] \times (\gamma _u^{2\omega }-\gamma _u^{2\omega *})\\ &{}+\left[ \overline{B}^1 - \sum _{\tilde{f}\in \tilde{\mathcal {F}}_{2}} {c^1_{\tilde{f}}\left( \sum _{u=1}^U\sum _{k=1}^K x^1_{u\tilde{f}k}\right) } -\sum _{u=1}^Uc_u^1(\gamma _{u}^1)\right] \times (\lambda ^1-\lambda ^{1*})\\ &{}+\sum _{\omega \in \Omega }\left[ {\overline{B}^{1}+} \overline{B}^{2\omega }- \sum _{\tilde{f}\in \tilde{\mathcal {F}}_{2}} {c^{1}_{\tilde{f}}\left( \sum _{u=1}^U\sum _{k=1}^K x^{1}_{u\tilde{f}k}\right) }-\sum _{u=1}^U c_u^1(\gamma _u^1)- \sum _{\tilde{f}\in \tilde{\mathcal {F}}_{2}} {c^{2\omega }_{\tilde{f}}\left( \sum _{u=1}^U\sum _{k=1}^K x^{2\omega }_{u\tilde{f}k}\right) }\right. \\ &{}\left. \qquad \qquad -\sum _{u=1}^U c_u^{2\omega }(\gamma _u^{2\omega })\right] \times (\lambda ^{2\omega }-\lambda ^{2\omega *})\ge 0 \end{aligned}}$$

35 $$\begin{aligned} \forall (X,\lambda ^{1},\lambda ^{2})\in \mathbb {K}^2, \end{aligned}$$

where

$$\mathbb {K}^2:=\Big \{(X,\lambda ^1,\lambda ^2)\in \mathbb {R}^{U(1+|\Omega |)[(G+F_3)K+1]}:(2)-(5);(7)-(8);(12)-(15);(17)-(18) \ \text{ hold }\Big \}.$$

## Numerical Examples

In this section, we provide some numerical examples to illustrate some key aspects of the model.

### Network Topology and Data

**Fig. 2:**
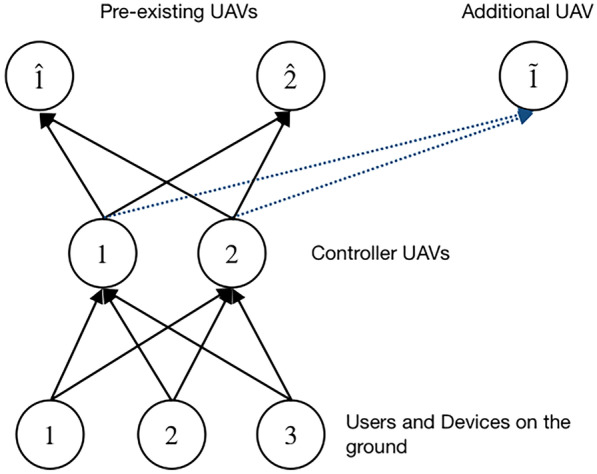
Network Topology for the Numerical Examples

As depicted in Fig. 2, we consider three users and devices on the ground ($G=3$) requiring a 5G service ($K=1$), two controller UAVs ($U=2$) which receive the service requests (from users and devices) and send them to the two UAVs belonging to the fleet at the highest level of the network ($\hat{F}_1=2$) where services are performed. We also analyze the possibility to add the capacity at each controller UAV and to add a new UAV to the fleet ($\tilde{F}_2=1$).

Moreover, in these examples we assume that 2 different scenarios (see [37] and [27]), $\omega _1=1$ and $\omega _2=2$, can occur with different probabilities:

- Case 1: $$p_{1}=0.8$$ and $$p_2=0.2$$;
- Case 2: $$p_{1}=0.2$$ and $$p_2=0.8$$;
- Case 3: $$p_{1}=1.0$$ and $$p_2=0.0$$.

Note that in Case 1 it is estimated that the first scenario has a higher probability of occurrence than the second scenario; vice versa in Case 2. Finally, in Case 3 it is estimated that only the first scenario can occur (indeed the probability that the second scenario occurs is zero).

We suppose the following general expressions for the cost functions, for all $g,u,\hat{f},\tilde{f}$:

$$\displaystyle c_{gu}^1=a_{gu}\left( \sum _{k=1}^Kx_{guk}^1\right) ^2+b_{gu}\left( \sum _{k=1}^Kx_{guk}^1\right) , \displaystyle c_{gu}^{2\omega }=a_{gu}\left( \sum _{k=1}^Kx_{guk}^{2\omega }\right) ^2+b_{gu}\left( \sum _{k=1}^Kx_{guk}^{2\omega }\right) ;$$

$$\displaystyle c_{uf}^1=d_{uf}\left( \sum _{k=1}^Kx_{ufk}^1\right) ^2+e_{uf}\left( \sum _{k=1}^Kx_{ufk}^1\right) , \displaystyle c_{uf}^{2\omega }=d_{uf}\left( \sum _{k=1}^Kx_{ufk}^{2\omega }\right) ^2+e_{uf}\left( \sum _{k=1}^Kx_{ufk}^{2\omega }\right) ;$$

$$\begin{aligned} \displaystyle c_{f}^{1(E)}=g_f\left( \sum _{u=1}^U\sum _{k=1}^K{x_{ufk}^1} \right) ^2+h_f\left( \sum _{u=1}^U\sum _{k=1}^Kx_{ufk}^1\right) , \end{aligned}$$

$$\begin{aligned} \displaystyle c_{f}^{{2\omega }(E)}=g_f\left( \sum _{u=1}^U\sum _{k=1}^K{x_{ufk}^{2\omega }} \right) ^2+h_f\left( \sum _{u=1}^U\sum _{k=1}^Kx_{ufk}^{2\omega }\right) ; \end{aligned}$$

$$\begin{aligned} \displaystyle c_{\tilde{f}}^1=i_{\tilde{f}}\left( \sum _{u=1}^U\sum _{k=1}^K x_{u\tilde{f}k}^1\right) ^2+j_{\tilde{f}}\left( \sum _{u=1}^U\sum _{k=1}^K x_{u\tilde{f}k}^1\right) , \end{aligned}$$

$$\begin{aligned} \displaystyle c_{\tilde{f}}^{2\omega }=i_{\tilde{f}}\left( \sum _{u=1}^U\sum _{k=1}^K x_{u\tilde{f}k}^{2\omega }\right) ^2+j_{\tilde{f}}\left( \sum _{u=1}^U\sum _{k=1}^Kx_{u\tilde{f}k}^{2\omega }\right) ; \end{aligned}$$

$$\begin{aligned} c_u^1=k_u(\gamma _u^{1})^2+l_u\gamma _u^{1},\;c_u^{2\omega }=k_u(\gamma _u^{2\omega })^2+l_u\gamma _u^{2\omega }. \end{aligned}$$

Observe that, as mentioned above, no constant term appears in these functions. Therefore, if there is no flow in a link of the network (from a user or device on the ground to a controller UAV or from a controller UAV to a UAV in the upper tier fleet), the cost of transmission in that link is zero. Similarly, if a UAV does not execute any service, the execution cost for that UAV is zero. The same for the cost needed for additional UAVs and capacities.

**Table 3:** Coefficients for the cost functions involved in the mathematical formulation

**Description**	**Numerical data**
$a_ {gu}$ and $b_{gu}$: Coefficients of the transmission cost of services from user *g* to the controller UAV *u* both in the first and second stage under scenario $\omega$ ($c_{gu}^1$, $c_{gu}^{2\omega }$)	$a_{11}=3$, $a_{12}=4$, $a_{21}=1$, $a_{22}=1$, $a_{31}=4$, $a_{32}=2$, $b_{11}=3$, $b_{12}=4$, $b_{21}=1$, $b_{22}=1$, $b_{31}=3$, $b_{32}=2$
$d_{uf}$ and $e_{uf}$: Coefficients of the transmission cost of the service requests from controller UAV *u* to any UAV $f\in \mathcal {F}_3$ both in the first and second stage under scenario $\omega$, ($c_{uf}^1$, $c_{uf}^{2\omega }$)	$d_{1\hat{1}}=2$, $d_{1\hat{1}}=2$, $d_{1\tilde{1}}=2$, $d_{2\hat{1}}=3$, $d_{2\hat{2}}=1$, $d_{2\tilde{1}}=2$, $e_{1\hat{1}}=2$, $e_{1\hat{1}}=2$, $e_{1\tilde{1}}=2$, $e_{2\hat{1}}=3$, $e_{2\hat{2}}=1$, $e_{2\tilde{1}}=2$
$g_{f}$ and $h_{f}$: Coefficients of the execution cost of requested services to the UAV $f\in \mathcal {F}_3$ both in the first and second stage under scenario $\omega$, ($c_{f}^{(E),1}$, $c_{f}^{(E),2\omega }$)	$g_{\hat{1}}=g_{\hat{2}}=g_{\tilde{1}}=1=h_{\hat{1}}=h_{\hat{2}}=h_{\tilde{1}}$
$i_{\tilde{f}}$ and $j_{\tilde{f}}$: Coefficients of the cost due to add a new UAV $\tilde{f}\in \tilde{\mathcal {F}}$ at the highest level of the network both in the first and second stage under scenario $\omega$ ($c_{\tilde{f}}^{1}$, $c_{\tilde{f}}^{2\omega }$)	$i_{\tilde{1}}=5=j_{\tilde{1}}$
$k_u$ and $l_u$: Coefficients of the cost due to add additional capacity to the controller UAV *u*, both in the first and second stage under scenario $\omega$ ($c_{u}^{1}$, $c_{u}^{2\omega }$)	$k_1=1$, $k_2=2$, $l_1=1$, $l_2=1$

For the numerical setting, we consider the coefficients for the cost functions involved in the formulation as in Table 3. Moreover, we select the parameters as follows:

$$\beta _1=50;\;\rho _1=100,\;\rho _2=100;\;s_1=1;\;S_{\hat{1}}=S_{\hat{2}}=4,\;S_{\tilde{1}}=7;$$

$$\overline{S}_1=\overline{S}_2=4;\;\overline{\gamma }_1^1=\overline{\gamma }_2^1=\overline{\gamma }_1^{2\omega }=\overline{\gamma }_2^{2\omega }=4;$$

$$R_{11}^1=R_{21}^1=R_{31}^1=2;\;R^{21}_{11}=6,\; R_{21}^{21}=10,\;R_{31}^{21}=0;\;R^{22}_{11}=3,\;R^{22}_{21}=4,\;R^{22}_{31}=3.$$

We have made the choice of this data assuming that the second user or device on the ground ($g=2$) is in a central position and is closest to both the controller UAVs, indeed, the coefficients of its transmission cost functions ($a_{gu}$ and $b_{gu}$) are the smallest: $a_{21}=b_{21}=a_{22}=b_{22}=1$. The first user or device on the ground ($g=1$) is closer to the first controller UAV, and it is the furthest away from the second one ($a_{11}=b_{11}=3,\;a_{12}=b_{12}=4$). On the contrary, the third user or device ($g=3$) is closer to the controller UAV $u=2$ than to the controller UAV $u=1$ ($a_{31}=4,\;b_{31}=3,\;a_{32}=b_{32}=2$). In a similar way, we are assuming that the second UAV belonging to the fleet, $\hat{f}=\hat{2}$ is geographically positioned in such a way that it is central and, hence, it is the closest one to both the controller UAVs, indeed, the coefficients of its transmission cost functions ($d_{uf}$ and $e_{uf}$) are the smallest: $d_{1\hat{2}}=e_{1\hat{2}}=d_{2\hat{2}}=e_{2\hat{2}}=1$. The first UAV belonging to the fleet, $\hat{f}=\hat{1}$, is closer to the first controller UAV than to the second ($d_{1\hat{1}}=e_{1\hat{1}}=2,\;d_{2\hat{1}}=e_{2\hat{1}}=3$). Moreover, we assume that the additional UAV $\tilde{f}=\tilde{1}$ can be positioned to be equally distant from both the controller UAVs ($d_{1\tilde{1}}=e_{1\tilde{1}}=d_{2\tilde{1}}=e_{2\tilde{1}}=2$). In this way we have represented the different geographical scenarios that can arise, and it is easy to understand how to represent and extend further real cases.

Since all the coefficients, $g_{f}$ and $h_f,\; \forall f\in \mathcal {F}_3$, are the same, in these numerical examples we are assuming that all the UAVs have the same unit execution cost but it is clear that this does not constitute a limit since it is sufficient to replace these coefficients with more appropriate ones.

Furthermore, all the costs, included the costs due to insert additional UAVs into the network and adding capacity to the controller UAVs are the same for both stages but we are assuming adding capacity to the second controller UAV is slightly more expensive (because $k_2=2$ while $k_1=1$).

Regarding the capacity limits, we suppose a difference only on the maximum execution capacity of the additional UAV, $S_{\tilde{1}}=7$, that is greater than the maximum execution capacities of the pre-existing ones ($S_{\hat{1}}=S_{\hat{2}}=4$). Observe that the total demand in Stage 1, when no disastrous event occurs, is equal to 6; while in Stage 2, that is when a disastrous event occurs, in both scenarios $\omega _1$ and $\omega _2$, the total demand $\displaystyle \sum _{g=1}^G\sum _{u=1}^UR_{gu}^{2\omega }$ is greater than that of the first stage. Particularly, in the first scenario there is a total demand equal to 16, while in the second scenario it is equal to 10. Therefore, Scenario 1 is more severe than Scenario 2. Moreover, we are assuming that in Scenario 1 the increase in demand is not constant, but actually decreases in one user or device and increases consistently in another user or device. On the other hand, in Scenario 2, the increase in demand is fairly uniform for all users and devices on the ground. Such data are in keeping with reality as disaster scenarios can be of different types: some have a zeroing of requests in one area and simultaneously an increase in other areas while other scenarios have a uniform increase in all areas.

We suppose two different situations: in the Situation 1 we assume that the maximum available budgets (for additional UAVs at the highest level of the network and the additional capacities at the controller UAVs) are the same for each stage and for each scenario (that is $\overline{B}^1=\overline{B}^{21}=\overline{B}^{22}=200$); instead, in Situation 2 we assume different maximum available budgets. Particularly, we presume that the budget for the first stage (when no disaster event occurs) is less than the available budgets for both the scenarios, $\omega _1$ and $\omega _2$, of the second stage and that the budget available for $\omega _1$, the most serious scenario, is greater than that for $\omega _2$ ($\overline{B}^1=50,\overline{B}^{21}=200,\overline{B}^{22}=120$).

### Optimal Solutions

For each of the 6 numerical examples (three cases and two situations), in Tables 4, 5 and 6 are reported the values of the optimal solutions for Stage 1, Stage 2 - Scenario 1 and Stage 2 - Scenario 2, respectively. We obtain these optimal solutions by solving the Variational Inequality (35) and are computed through the Euler Method (see [38] for a detailed description) using the MATLAB program on an HP laptop with an AMD compute cores 2C+3G processor, 8 GB RAM.

**Table 4:** Optimal solutions: Stage 1

	**Situation 1:**			**Situation 2:**		
	$\overline{B}^1=\overline{B}^{21}=\overline{B}^{22}=200$			$\overline{B}^1=50,\overline{B}^{21}=200,\overline{B}^{22}=120$		
	**Case 1:**	**Case 2:**	**Case 3:**	**Case 1:**	**Case 2:**	**Case 3:**
**Variables**	$p_1=0.8$	$p_1=0.2$	$p_1=1$	$p_1=0.8$	$p_1=0.2$	$p_1=1$
	$p_2=0.2$	$p_2=0.8$	$p_2=0$	$p_2=0.2$	$p_2=0.8$	$p_2=0$
$x_{111}^1$	2.74	1.49	2.70	1.41	1.54	1.37
$x_{121}^1$	2.26	0.76	2.30	0.59	0.87	0.63
$x_{211}^1$	3.30	1.94	3.31	2.05	1.87	2.07
$x_{221}^1$	2.70	1.80	2.69	1.95	1.71	1.93
$x_{311}^1$	0.75	0.57	0.77	0.54	0.58	0.56
$x_{321}^1$	1.25	1.43	1.23	1.46	1.42	1.44
$x_{1\hat{1}1}^1$	2.45	2.25	2.45	2.34	2.23	2.34
$x_{1\hat{2}1}^1$	1.90	1.75	1.89	1.66	1.77	1.66
$x_{2\hat{1}1}^1$	1.55	1.75	1.55	1.66	1.77	1.66
$x_{2\hat{2}1}^1$	2.10	2.25	2.11	2.34	2.23	2.34
$x_{1\tilde{1}1}^1$	2.44	0.00	2.44	0.00	0.00	0.00
$x_{2\tilde{1}1}^1$	2.56	0.00	2.56	0.00	0.00	0.00
$\gamma _{1}^1$	2.80	0.00	2.78	0.00	0.00	0.00
$\gamma _{2}^1$	2.20	0.00	2.22	0.00	0.00	0.00

**Table 5:** Optimal solutions: Stage 2 - Scenario 1

	**Situation 1:**			**Situation 2:**		
	$\overline{B}^1=\overline{B}^{21}=\overline{B}^{22}=200$			$\overline{B}^1=50,\overline{B}^{21}=200,\overline{B}^{22}=120$		
	**Case 1:**	**Case 2:**	**Case 3:**	**Case 1:**	**Case 2:**	**Case 3:**
**Variables**	$p_1=0.8$	$p_1=0.2$	$p_1=1$	$p_1=0.8$	$p_1=0.2$	$p_1=1$
	$p_2=0.2$	$p_2=0.8$	$p_2=0$	$p_2=0.2$	$p_2=0.8$	$p_2=0$
$x_{111}^{21}$	2.00	3.37	2.07	3.68	3.29	3.68
$x_{121}^{21}$	1.00	2.38	0.93	2.32	2.29	2.32
$x_{211}^{21}$	3.00	4.35	2.93	4.32	4.42	4.32
$x_{221}^{21}$	3.00	3.91	3.07	3.68	4.00	3.68
$x_{311}^{21}$	0.00	0.00	0.00	0.00	0.00	0.00
$x_{321}^{21}$	0.00	0.00	0.00	0.00	0.00	0.00
$x_{1\hat{1}1}^{21}$	2.51	2.55	2.52	4.00	2.55	4.00
$x_{1\hat{2}1}^{21}$	1.98	2.11	1.97	3.67	2.11	3.56
$x_{2\hat{1}1}^{21}$	1.49	1.45	1.48	0.00	1.45	0.00
$x_{2\hat{2}1}^{21}$	2.02	1.89	2.03	0.33	1.89	0.44
$x_{1\tilde{1}1}^{21}$	0.51	3.06	0.51	0.33	3.06	0.44
$x_{2\tilde{1}1}^{21}$	0.49	2.94	0.49	5.67	2.94	5.56
$\gamma _{1}^{21}$	1.00	3.71	1.00	4.00	3.71	4.00
$\gamma _{2}^{21}$	0.00	2.29	0.00	2.00	2.29	2.00

**Table 6:** Optimal solutions: Stage 2 - Scenario 2

	**Situation 1:**			**Situation 2:**		
	$\overline{B}^1=\overline{B}^{21}=\overline{B}^{22}=200$			$\overline{B}^1=50,\overline{B}^{21}=200,\overline{B}^{22}=120$		
	**Case 1:**	**Case 2:**	**Case 3:**	**Case 1:**	**Case 2:**	**Case 3:**
**Variables**	$p_1=0.8$	$p_1=0.2$	$p_1=1$	$p_1=0.8$	$p_1=0.2$	$p_1=1$
	$p_2=0.2$	$p_2=0.8$	$p_2=0$	$p_2=0.2$	$p_2=0.8$	$p_2=0$
$x_{111}^{22}$	0.00	1.61	0.00	1.76	1.55	0.00
$x_{121}^{22}$	0.00	1.13	0.00	1.24	1.04	0.00
$x_{211}^{22}$	0.00	1.37	0.00	1.24	1.45	0.00
$x_{221}^{22}$	0.00	0.88	0.00	0.76	0.96	0.00
$x_{311}^{22}$	1.09	1.01	0.00	1.00	1.00	0.00
$x_{321}^{22}$	1.91	1.99	0.00	2.00	2.00	0.00
$x_{1\hat{1}1}^{22}$	0.48	0.00	0.00	2.25	2.20	0.00
$x_{1\hat{2}1}^{22}$	0.61	0.53	0.00	1.75	1.80	0.00
$x_{2\hat{1}1}^{22}$	0.33	0.00	0.00	1.75	1.80	0.00
$x_{2\hat{2}1}^{22}$	1.58	0.47	0.00	2.25	2.20	0.00
$x_{1\tilde{1}1}^{22}$	0.00	3.47	0.00	0.00	0.00	0.00
$x_{2\tilde{1}1}^{22}$	0.00	3.53	0.00	0.00	0.00	0.00
$\gamma _{1}^{22}$	0.00	0.00	0.00	0.00	0.00	0.00
$\gamma _{2}^{22}$	0.00	0.00	0.00	0.00	0.00	0.00

**Fig. 3:**
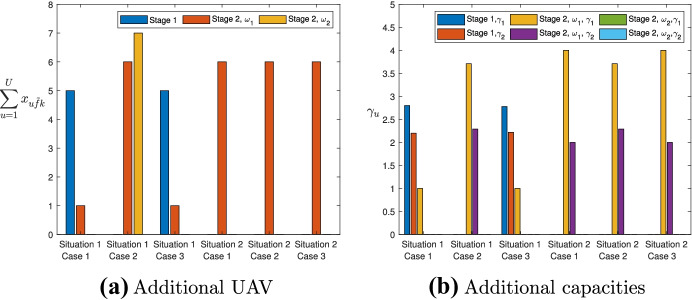
Main optimal solutions

The main optimal solutions are also reported in Fig. 3, in which we have indicated the use (or not) of the additional UAV, graphing the sum of the optimal variables relating to the flows toward the additional UAV ($\displaystyle \sum _{u=1}^U x_{u\tilde{f}k}$) in Fig. 3a and the optimal variables relating to the additional capacities of each controller UAV ($\gamma _u$) in Fig. 3b.

### Analysis of the Results

We highlight that in Stage 1 of the first situation, Case 1 (second column of Table 4), 7 more services are performed (6 was the total demand requested and 13 was the one executed) which are provided at Stage 2. To execute such services, both the additional capacities and additional UAVs are used at the first stage, while, additional capacity of only one controller UAV is used at the first scenario of Stage 2 (whose severity is greater), and no additional UAVs and additional capacities are used at Stage 2, Scenario 2.

When the maximum budget for additional UAVs and additional capacities is tight (Situation 2) we get a different optimal solution where no additional UAVs and additional capacities are used in Stage 1; while they are used (for all the UAVs) in Scenario 1 of Stage 2 (see Table 5 and Fig. 3).

We also emphasize that, unlike Case 1 (Situation 1), in Case 2, since the probability of occurrence of the most serious disaster scenario is lower (0.2 rather than 0.8), in Stage 1 no additional UAVs and capacities are used while additional UAVs are used in both scenarios of Stage 2 and the additional capacities of both the controller UAVs in the first scenario of Stage 2 are used.

Moreover, in Scenario 2 of Case 2, it is convenient to use the additional UAVs in Situation 1 (in which we have the necessary budget), instead, in Situation 2 we cannot use the additional UAVs because the available budget is not enough to add new UAVs (see columns 3 and 6 of Table 6).

Case 3 is very similar to Case 1, but the second scenario cannot happen (as $p_2 = 0$), therefore, all optimal solutions related to the second scenario are null.

Finally, we observe that, unlike Cases 1 and 3 of Situation 1, in the other cases and situations, in the first stage fewer more services are performed (only 2 more services); this is explained because either there are not enough budgets (Situation 2) or the disastrous scenario, in which the demand is lower, is more likely to occur (Case 2).

#### Additional Discussion On the Results

From the analysis of the obtained optimal solutions, it is possible to deduce some key findings that we will summarize here.

The results reveal that the management of both the preparation and the response phases has a significant impact when a disaster scenario occurs. In this paper we firstly analyzed the effects that scenarios of different severity could cause. Particularly, we assume that the severity of each scenario is represented by an unexpected and sudden increase in demand which can be more or less intense. Therefore, we supposed that in both the considered disaster scenarios the service requests increase, and that the first scenario is more severe, since the total quantity of requests in $\omega =1$ increases dramatically, especially is some areas (while in other areas the demand is zeroed), meanwhile, in the second scenario, requests for services increase fairly equally for all areas. So, even if with only two scenarios, we represented the most common types of demand changes.

We also underline that each of these scenarios can occur with different probabilities. Therefore, in our numerical examples we have analyzed three different cases, in which such occurrence probabilities vary. Particularly, in Case 1 we supposed that the more severe scenario has a higher occurrence probability than the other scenario; on the contrary, in Case 2 we assumed that the scenario with higher probability is the softer one. Finally, in Case 3 we analyzed the situation in which only a scenario can occur.

The main aim of the model proposed in this paper is to optimize the management of the pre-existing and additional resources. Therefore, in our illustrative examples we investigated the behavior of the service provider (that seeks to maximize his total profit and to minimize the expected loss related to a possible unmet demand), paying particular attention to the possibility that he has to use or not additional resources. More specifically, we analyzed the obtained optimal results, observing if it is convenient, for the service provider, to use additional resources that means to put into flight new UAVs and, hence, to add them to the network and/or to further increase the capabilities of pre-existing controller UAVs (at the first and/or second stage), making possible to satisfy a greater number of requests.

Another fundamental aspect, especially in a disaster situation, not to be underestimated, is represented not only by the costs needed to add such resources, namely the costs due to add new UAVs at the highest level of the network and the costs due to add capacity to the controller UAVs, but also by the maximum available budget for additional resources: there exists a budget constraint which states that the overall cost for additional UAVs at the highest level of the network and additional capacities at the controller UAVs must not exceed the budget limit, $\overline{B}^1$ or $\overline{B}^{2\omega }$ (in the first or second stage, respectively). Therefore, in our illustrative numerical experimentation we also analyzed some managerial insights obtained by not only the aforementioned cases but also by two different situations in which the maximum budget limits ($\overline{B}^1$ and $\overline{B}^{2\omega }$) vary.

Therefore, from the overall analysis of the examples, cases and situations that we have examined, and of the optimal solutions we found and reported above, we can deduce some key aspects that can provide support in managerial decisions.

We observed that during the preparation phase (Stage 1), that is when no disastrous scenario has yet occurred, it may be convenient to use additional resources or not. Indeed, in the case in which the scenario with the highest probability is the least serious one (Case 2), it is not necessary to use the additional resources and, hence, there is no cost to add UAVs or further capacities. On the contrary, it is more convenient to use both the additional UAVs and the additional capacities, when the most serious scenario (the one with the highest total demands) has a high probability of occurrence (cases 1 and 3), in order to satisfy the greatest number of requests.

It is important to point out that, as mentioned above, the budget limit assumes a fundamental role, in fact it should be noted that when the available budget is limited, the additional resources cannot be used (see Situation 2, Stage 1). Similarly, when the second scenario $\omega =2$ is the one with higher probability (Case 2), if the budget is enough, it is convenient to use additional UAVs. Indeed in Situation 1 ($\overline{B}^{22}=200$) they are used, while in Situation 2 ($\overline{B}^{22}=120$) they are not used. Therefore, it is clear that our model can also be used for simulations on the budgets by the service providers to estimate and establish if increasing the budget is suitable or not.

Further considerations can be expressed by comparing the results of the different stages and cases. When the most serious scenario has a high probability of occurrence (cases 1 and 3) and when the budget is enough, we have already observed that both the additional types of resources are used in Stage 1. In this situation, in stage 2, under scenario 1 it is convenient to use the additional UAVs, but to add capacity to just one of the controller UAVs while under scenario 2 it is not necessary to use additional resources (because the provider has fewer requests). If, instead, we are in Situation 2, where the budget was not sufficient (and therefore no additional resources were used in Stage 1), we obtain that in Stage 2, under Scenario 1 (the most serious), it is convenient to use all the additional resources, as is also if the less serious scenario is more likely (Case 2). Therefore, it is easy to guess that the proposed model is also useful for investigating the consequences of scenarios and their probability of occurrence, in the different stages, with a particular reference to the suitable use of additional resources.

## Conclusions

In this paper, we proposed a two-stage stochastic optimization model describing the provision of services in case of a disaster scenarios. The uncertainty of a such events, their severity and the unpreparedness of service providers could make challenging, expensive and time consuming the provision of services in case of disaster. Hence, to mitigate the consequences of a disaster advent, it is of fundamental importance to provide mathematical models that can allow service providers to make predictions on possible disastrous scenarios and act in such a way as to minimize the loss associated with a possible unsatisfied demand. This challenges led us to the two-stage optimization model described in this paper, which aims to maximize the profits associated with the provision of 5G services and, simultaneously, minimize the expected loss associated with a sudden growth in request. A variational approach was used as a tool to obtain the optimal solution of some numerical examples. The obtained results show that not only the optimal distribution of flows, but also the optimal management of any additional resources assumes a main role, together with the maximum budget available for these additional resources and the different probabilities that disastrous scenarios (more or less serious) will occur. From the optimal solutions it emerges that the model turns out to be efficient, in case of prediction of one or more disastrous events (even different from each other), since it is able to help the services providers both in the preparation phase (Stage 1) and in the response phase (Stage 2). Indeed, it is possible to start preparing for the disastrous event even during Stage 1, always satisfying all constraints, including the nonlinear budget constraints. Summarizing the results obtained from the numerical examples, we can highlight the following key findings:

- We have predisposed different scenarios able to represent the common situations that can occur in the event of a disaster: increase in requests in a fairly evenly distributed manner or movement of requests from one area to another;
- We have conceived some cases in which the probabilities of occurrence of the scenarios vary;
- We have simulated different situations in which the maximum budgets available for the costs due to add/use additional resources (additional UAVs and additional capacities) vary;
- We have deduced the main features of the optimal behavior of service providers, regarding the convenience or otherwise on the use of additional resources. Such deductions provide us with some managerial insights and the results allow the service providers to carry out simulations and to make optimal decisions.

The model is very suitable to be applied to real-life situations, because all its components (variables, objective function and constraints, the cost functions) allow us to represent reality. Despite this, the difficulties we expect to face when applying the model to real-life situations are given by some computational aspects due to the large-scale instances and iterative computational times required to solve the problem. Therefore, in future works, another interesting research would be solving large-scale examples using real data and alternative computational methods to test the validity of our model. Furthermore, we are going to study and analyze a new heuristic approach and a new model with 3 stages.

This proposed model could also be useful for providing other services or things in emergency situations.

## Data Availability

All data generated or analyzed during this study are included in this published article.
